# High Prevalence of HPV 51 in an Unvaccinated Population and Implications for HPV Vaccines

**DOI:** 10.3390/vaccines10101754

**Published:** 2022-10-20

**Authors:** Sarah J. Bowden, Laura Burney Ellis, Maria Kyrgiou, Alison N. Fiander, Samantha Hibbitts

**Affiliations:** 1 IRDB, Imperial College London, London W12 0NN, UK; 2 School of Medicine, Cardiff University, Cardiff CF14 4XN, UK

**Keywords:** HPV, human papillomavirus, vaccine, type-replacement, cervical, cross-protection

## Abstract

Human papillomavirus (HPV) is detected in 99.7% of cervical cancers. Current vaccines target types 16 and 18. Prior to vaccination implementation, a prospective cohort study was conducted to determine baseline HPV prevalence in unvaccinated women in Wales; after HPV16 and HPV18, HPV 51 was found to be most prevalent. This study aimed to re-assess the unexpected high prevalence of HPV 51 and consider its potential for type-replacement. Two hundred HPV 51 positive samples underwent re-analysis by repeating the original methodology using HPV 51 GP5+/6+ PCR-enzyme immunoassay, and additionally a novel assay of HPV 51 E7 PCR. Data were correlated with age, social deprivation and cytology. Direct repeat of HPV 51 PCR-EIA identified 146/195 (75.0%) samples as HPV 51 positive; E7 PCR identified 166/195 (85.1%) samples as HPV 51 positive. HPV 51 prevalence increased with cytological grade. The prevalence of HPV 51 in the pre-vaccinated population was truly high. E7 DNA assays may offer increased specificity for HPV genotyping. Cross-protection of current vaccines against less-prevalent HPV types warrants further study. This study highlights the need for longitudinal investigation into the prevalence of non-vaccine HPV types, especially those phylogenetically different to vaccine types for potential type-replacement. Ongoing surveillance will inform future vaccines.

## 1. Introduction

Globally, cervical cancer is the second most common cancer in women and a leading cause of cancer mortality [1]. Human papillomavirus (HPV) infection is known to be a necessary and causal factor for the development of cervical cancer [1,2] and is found in 99.7% of invasive cervical carcinomas [3,4]. HPV is highly prevalent and considered the most common sexually transmitted agent worldwide [1]. The lifetime chance of HPV infection is estimated to be 80% [5]. In the majority of women, this infection is transient and will not result in cancer [3]. However, in a small number of cases, HPV infection persists, and invasive cervical carcinoma develops.

Over 200 HPV genotypes have now been identified, of which about 14 HPV types are known to be oncogenic and classified as high-risk (HR-HPV) [4,5,6]. HPV type-specific prevalence varies globally, however, types 16 and 18 are generally the most prevalent and are thought to be responsible for at least 70% of invasive cervical carcinoma [4,7]. These types therefore became the target of the first prophylactic HPV vaccines. Baseline study of the type-specific prevalence of HPV is important for assessment of the impact of prophylactic HPV vaccination and for type-replacement monitoring.

In September 2008, the national HPV vaccination programme was implemented in the UK, offering Cervarix (an HPV-16/18 AS04-adjuvanted vaccine, GlaxoSmithKline Biologicals, United Kingdom) to all girls between ages 12–13. Maximum efficacy is expected in women who have not been previously exposed to HPV [8]. In September 2012, the vaccine offered by the UK national programme was changed to Gardasil (quadrivalent vaccine active against HPV-16/18/6/11, Merck, Germany) to offer additional protection against low-risk HPV types responsible for genital warts. The full extent of cross-protection against non-vaccine types is yet unknown. Similarly, type-replacement of vaccine types with non-vaccine types through ecological pressure is a theoretical risk [9,10].

Between March 2009 and November 2010, the HPV Research Group at Cardiff University in collaboration with Cervical Screening Wales conducted a prospective cohort study of women at entry to the cervical screening programme. A total of 14,128 pseudo-anonymous liquid-based cytology (LBC) samples were collected from women aged 20–22 years, who had not been offered the HPV vaccine. Preliminary analysis showed that in single infections HPV 16 was the most prevalent (32.0%) followed by HPV 18 (11.2%) and HPV 51 (10.8%) [11]. HPV 51 prevalence among multiple infected samples was also high at 18.4%. HPV genotype prevalence is heterogeneous worldwide. However, this high proportion of HPV 51 is not a common finding of previous prevalence studies and was unexpected [1,3,12]. High-risk human papillomavirus GP5+/6+ PCR-enzyme immunoassay test (GP5+/6+ PCR EIA) is considered a gold standard assay with high sensitivity and specificity [13]. However, in genotyping, several issues are known to cause false positives including contamination during sample processing and cross-hybridisation during HPV typing [14]. The finding of a high prevalence of HPV 51 was unexpected and had not previously been found in Wales. Additionally, HPV 51 is not directly covered by any current vaccine. Therefore, we aimed to re-assess this unanticipated finding of the initial samples, in order to rule out the possibility of contamination, or cross-hybridisation of HPV types within assays and confirm whether the prevalence of HPV 51 found was truly high. Through retesting a selection of samples and additionally using a novel assay for validation, and HPV sequencing, we aimed to confirm the prevalence of HPV 51 previously observed and importantly, discuss implications for HPV vaccines.

## 2. Materials and Methods

**Figure 1:**
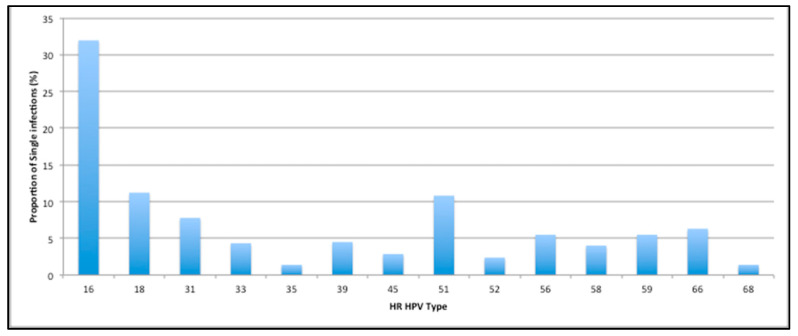
Proportion of single infections in the original cohort study.

From the original prospective cohort of 14,128 pseudo-anonymous LBC (BD SurePath, Source Bioscience, UK) samples from unvaccinated women [11], aged 20–22 years all available samples testing positive for HPV 51 were identified (*n* = 321) (Figure 1). Samples were eligible if the woman was attending her first cervical smear and had an adequate cytology result available. Samples were collected at time of patient presentation, over 16 months, and stored separately, negating the risk of contamination. The study was approved by Dyfed Powys Local Research Ethics Committee (08/WMW01/69). LBC samples (Thinprep) were processed by the Cytology Laboratory according to the British Society of Clinical Cytology guidelines [15]. Residual cell pellets were re-suspended in the alcohol-based liquid and processed by the HPV Laboratory, Cardiff University. In all, 132 samples were single infections of HPV 51 (singles) whilst 189 samples tested positive for HPV 51 and other HR HPV strains (multiples). In this study we chose to re-analyse 200 samples; the first 100 single and 100 multiple samples positive for HPV 51. The population characteristics for the selected samples are shown below (Table 1). In this study, DNA was re-extracted from the initial stored sample, according to the original methodology, to exclude the risk of contamination [11] (Text Supplement S1 in Supplementary Materials). Extracted DNA used in retesting was quantified using NanoDrop [16] to assess quality and quantity of DNA for sequencing. A total of 1200 ng of sample DNA in 30 microlitres of purified water was then sent for HPV 51 E7 sequencing. A PCR for the human β- globin gene was performed on every DNA sample to determine extraction efficiency as previously described [12]. All samples were re-analysed using the following steps:

(a) Repeat of original GP5+/6+ PCR EIA (gold standard): the GP5+/6+ HPV PCR-ELISA method [17] was performed on all specimens in a 96-well format with minor modifications (Text Supplement S2 in Supplementary Materials).
(b) HPV 51 type-specific E7 Linear PCR followed by E7 Nested PCR (Text Supplements S3 and S4 in Supplementary Materials).
(c) Repeat of Discordant Results: where there was discordance between GP5+/6+ PCR EIA and E7 PCR results, samples were retested using both methods. As we had previously observed increased specificity with Hotstar Taq (Supplement Figures S1 and S2), all samples were repeated with Hotstar Taq (Text Supplement S5 in Supplementary Materials).
(d) HPV sequencing: to confirm the specificity of the E7 PCR method we sent a selection of positive samples for sequencing, analysis was undertaken using 4 peaks^®^ software and a BLAST^®^ search using megablast.

Statistical analysis was performed on all samples that were β-globin or HPV 51 positive. HPV 51 GP5+/6+ PCR EIA positive samples were defined as samples that tested positive in the original cohort and also positive in either the 2nd or 3rd repeat ELISA i.e., at least 2 of 3 positive results. HPV 51 E7 PCR positive samples were defined as samples that tested positive in the E7 PCR test of all samples using the Hotstar Taq method. Data on cytology, age and social deprivation status for each sample were aligned with repeated HPV test results using GP5+/6+ PCR EIA and E7 PCR methods. Social deprivation was estimated by linking postcodes to the Welsh Index of Multiple Deprivation which describes levels of deprivation across Wales, described in quartiles with higher scores indicating greater deprivation [18]. Association between tests was calculated using Kappa analysis. Fisher’s exact test was used to calculate p-values where appropriate.

## 3. Results

Five samples were found to be β-globin PCR and HPV 51 negative and were deemed inadequate and excluded from statistical analysis (Supplement Figure S3). One of these samples was single infection, four were multiple. Therefore, 99 singles and 96 multiples totalling 195 samples were included for final analysis.

**Figure 2:**
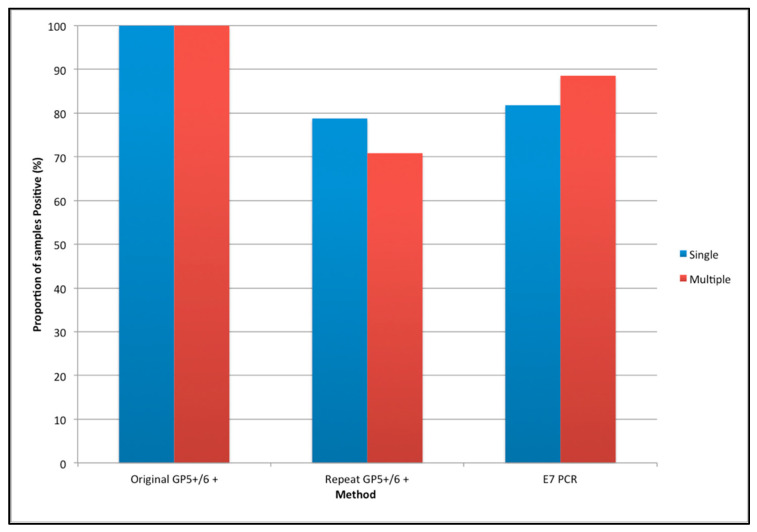
Proportion of samples testing HPV 51 positive by test: singles and multiples.

**Figure 3:**
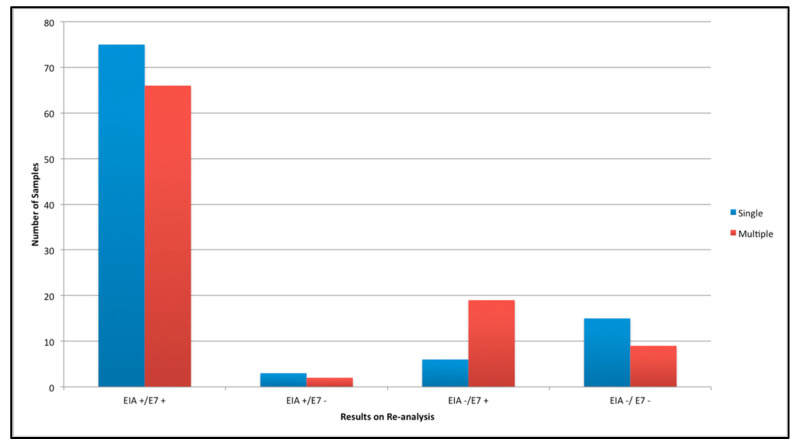
HPV 51 results from re-analysis with GP5+/6+ PCR EIA and E7 PCR (E7): singles and multiples.

Re-analysis using GP5+/6+ PCR EIA found that 146 of 195 samples (75.0%) tested HPV 51 positive. Of these HPV 51 positives, 78 were singles and 68 multiple infected samples. On re-analysis of the samples using E7 HPV 51 PCR it was found that 166/195 (85.1%) of samples tested positive. Of these, 81 were singles and 85 were multiples (Figure 2). Five samples tested positive by repeat GP5+/6+ PCR EIA and negative by E7 PCR. Twenty-five samples tested positive by E7 PCR and negative by repeat GP5+/6+ PCR EIA (Figure 3). Kappa analysis showed there was a moderate agreement between the repeat GP5+/6+ PCR EIA and E7 PCR tests (κ = 0.527 (95% CI 0.384 to 0.670)). Results were stratified by cytology, age and Social Deprivation Score (SDS). HPV 51 negative samples on repeat GP5+/6+ PCR EIA, were more commonly of a low cytological grade (Supplement Figure S4). The number of HPV 51 negative samples decreased as grade increased. No moderate or severe cytology samples retested negative for HPV 51 using E7 PCR. One severe but no moderate samples retested negative for HPV 51 using GP5+/6+. There was no observed difference between results stratified by age or SDS (Supplement Figures S5 and S6). Fragment sizes ranged from 94–219 bp. We compared the type-prevalence seen in all multiples with the type-prevalence seen in multiples which tested negative on reanalysis by GP5+/6+ PCR EIA or E7 PCR (Supplement Figure S7). The proportion of HPV 16 appeared to be higher in samples retesting negative by E7 PCR compared to GP5+/6+ PCR EIA negatives and all multiples. However, this was not found to be statistically significant (*p* = 0.4094 and *p* = 0.3016, respectively). Seven samples were sent for sequencing to confirm the specificity of our E7 51 PCR assay (Supplement Figure S8). Five sequences were found to be valid. There was no alignment with any known human sequence. Four known HPV genome alignments were seen for each of the five samples sent: the closest alignment was a 99% match to the known HPV 51 E7 region and three alignments of an 85–89% match to HPV 82 E7 region were also observed.

## 4. Discussion

The high prevalence of HPV 51 observed in a prospective cohort of unvaccinated women entering the cervical screening programme in Wales was unexpected and warranted further investigation. We repeated testing of samples with methods of enhanced specificity and developed a novel E7 type-specific PCR specific to HPV 51 in order to validate standard GP5+/6+ PCR EIA assays. As cross-hybridisation is likely to occur more frequently in samples infected by multiple HPV types, we analysed subgroups of single and multiple infections separately. In total, 75% of samples were still positive for HPV51 with repeat testing, this rose to 85.1% on retesting with a E7 PCR assay. Samples of high-grade cytology were most likely to retest positive and samples of negative cytology were most likely to retest negative. We found that multiples were more likely than singles to test negative by repeat GP5+/6+ PCR EIA but tested positive by the more specific E7 PCR method. Sequencing of positive samples for HPV51 E7 showed a 99% match across five samples suggesting excellent specificity. There was no significant association seen for age or SDS. Overall, these results suggest a low chance of cross-hybridisation and that a truly high prevalence of HPV51 exists in this cohort. However, intra-assay variation rates in the standard GP5+/6+ PCR EIA method were not insignificant and E7 PCR method may be a more reliable tool when assessing for cross-hybridisation in HPV assays.

Prevalence of HPV types varies greatly worldwide [19,20,21]. Our results have confirmed that a high HPV 51 prevalence exists in unvaccinated women aged 20–22 in Wales. A high proportion of HPV 51 has recently been seen in several prevalence studies undertaken in Scotland [22,23,24] and small studies of both HIV positive [25] and negative women in Sao Paulo, Brazil [26], in addition to studies in China [27,28], Iran [29] and Hungary [30]. However, in global meta-analyses of women with both cancerous and normal cytology, undertaken in 2011 and 2010, respectively, HPV 51 did not emerge as a highly prevalent genotype [19,20]. This raises the questions of whether HPV 51 is a significant genotype in other unknown areas and whether prevalence has increased since previous studies.

Multivalent vaccines, including the eight most prevalent HPV types, are anticipated to increase protection to >90% of cervical cancers based on studied populations in economically developed countries [31,32]. However, the majority of the world is understudied in terms of HPV type-specific prevalence. Non-vaccine types of significant prevalence in geographical regions include HPV 39, 52, 68 and 81. HPV 52 and 58 were found to be the most prevalent types in a study in Ghana [33] and HPV 52 was the most prevalent type in a recent study in Malaysian women [34]. In addition to HPV 52, HPV 68 and HPV 81 have found to be prevalent in China [35]. HPV 39, in addition to HPV 51, was found to be of significant prevalence in a post hoc analysis of unvaccinated and vaccinated populations in Finland [36]. In a prevalence study in Western Mexico, genotypes 59, 66, 52, 39 and 56 were found to be significant, in addition to HPV 51 [37]. HPV 51 belongs to the A5 species, which is phylogenetically different to the A9 and A7 species of HPV 16 and 18, respectively. The majority of the current evidence, including a randomised controlled trial containing 17,000 women, suggests that current licensed vaccines provide some degree of cross-immunity and protection against phylogenetically similar HR HPV types, but limited cross-protection against phylogenetically different but clinically important HR HPV types, including HPV 51 [2,6,38,39,40]. One randomised controlled trial initially observed that some cross-protection exists for HPV51 following vaccination [2], however, once co-infection with HPV16 and HPV18 was adjusted for, this effect was removed. Particularly given the phylogenetically different nature of HPV 51, further investigation into its prevalence is necessary to determine the need for its inclusion in any future multivalent vaccine.

As yet, it is unknown whether type-replacement will occur in the long-term following prophylactic vaccination. One important element in the likelihood of type-replacement is whether HPV types are synergistic, unrelated or competitive in their infection of the host and also on the cross-immunity of the vaccine used [41,42]. While one epidemiological modelling study suggested that type-replacement may occur [42], this was based on a theoretical model designed to mirror a real-world prevalence survey, rather than high-quality real-life data. Another study saw an increase in seroconversion to HPV33 with age, and concluded that HPV may compete for host infection and that a reduction in vaccine-types may lead to an ecological niche for the increase in non-vaccine types, but numbers of HPV33 positive women in the study were relatively small; 44 in the baseline seronegative group, and 56 in the baseline HPV16 or HPV18 positive group [43]. One study examining 1300 HPV-associated cancers [44] suggested infection by different HPV types is unrelated and independent. However, overall evidence, including data from randomised controlled trials, cohort studies and mathematical models, have suggested HPV types are synergistic, meaning any vaccine which decreases the prevalence of vaccine-types will lead to a reduction in infections by other types [38,39,45,46].

Analysis of pre- and post-vaccine populations has allowed monitoring of cross-protection of vaccines and has not shown convincing evidence of type-replacement thus far [47,48,49,50]. In a pooled analysis of two randomised control trials, including over 20,000 women four years after vaccination, there was no evidence of type replacement [51]. Similarly, several studies show convincing evidence for cross-protection [52], particularly of types phylogenetically related to 16/18 [48]. A recent systematic review including 19 studies observed that this effect may reduce with time [53], possibly because the current vaccines only induce priming of antibodies of non-vaccine types, suggesting the need for long-term follow-up of vaccinated cohorts.

The significance of a high prevalence of HPV 51 for cervical cancer also depends on other factors including the carcinogenicity of type 51. HPV 51 has been classified as definitely carcinogenic since 2005 but is thought to be less carcinogenic than HPV 16 and 18 [54,55]. HPV 51 is currently thought to account for around 1% of cervical cancer [56]. Cancer caused by HPV 51 may not be protected against under current vaccination schemes, however, overall HPV51 contributes to a small number of cancers. HPV primary testing was introduced into the NHS Cervical Screening Programme for England in April 2011 [57], current assays contain HPV51. The high prevalence of HPV 51 warrants the recommendation that type 51 is included in any type-specific HPV test used in clinical practice.

The results of this study were confined to the limitations of the assays used. Our results show variability in GP5+/6+ PCR EIA assays consistent with other laboratories. Contamination could have occurred during original testing, in probe synthesis or during setup of the HPV 51 plasmid. Viral aerosol in the laboratory could also have led to contamination. Variation in setup may have contributed to false positive or negative results; the reproducibility of GP5+/6+ PCR EIA has previously been found to be around 86% [58]. Where resources are not a limiting factor, every test should be repeated in triplicate to ensure reproducibility. Due to resource and time constraints not all samples that tested positive for HPV 51 in the original cohort were retested. In all, 200 of 321 samples were selected and results are therefore limited by sample size. To confirm the specificity of our E7 PCR method we sent a selection of positive samples for sequencing. Sequencing showed a 99% match to the known HPV 51 E7 sequence in all five samples. There was also a close match (85–89%) to HPV 82, which is a low prevalence HR HPV known to be phylogenetically similar and of the same A5 species as HPV 51 [59,60]. Type 82 was not included in type-specific assay and therefore the prevalence in the original population is unknown. However, the genetic sequences of 82 and 51 are sufficiently different to make the chance of HPV 82 being detected through E7 51 PCR unlikely. Sequencing of a larger sample size would allow for a firmer conclusion. Although HPV DNA assays offer a high sensitivity, specificity is lower. E6 and/or E7 oncoprotein testing has been suggested as a method to screen for cervical cancer in resource-poor settings [61], or as a triage tool [62,63] in HPV positive women. Our study suggests that these warrant further investigation.

## 5. Conclusions

Our results confirm a high prevalence of HPV 51 in unvaccinated young women in Wales. Currently evidence suggests it is unlikely that type-replacement will occur in the initial phases following prophylactic HPV vaccination. However, type-specific prevalence remains unknown in many geographical populations, monitoring of HPV type specific prevalence with vaccine introduction, with HPV assays of high sensitivity and specificity, will be important for measuring the impact of multivalent HPV vaccines and the movement towards global elimination of cervical cancer.

## Figures and Tables

**Table 1:** Study population characteristics including by subgroup of single infected HPV51 samples and multiple infections of HPV51 and other HPV type.

	Single (*n*)	Multiple (*n*)
**Age (years)**		
20	62	65
21	23	18
22	15	17
**SDS**		
NA	4	3
Q1	17	20
Q2	18	16
Q3	23	17
Q4	20	23
Q5	18	21
**Cytology**		
Negative	53	47
Borderline	22	26
Mild	23	22
Moderate	2	2
Severe	0	3

Abbreviations: SDS: social deprivation score, NA: not available.

## Data Availability

There is no further supporting data for this study.

## Supplementary Materials

The following are available online at https://www.mdpi.com/article/10.3390/vaccines10101754/s1, Supplement S1: Protocol for DNA Extraction, Supplement S2: Protocol for GP5+/6+ PCR EIA, Supplement S3: Protocol for Linear E7 PCR, Supplement S4: Protocol for Nested E7 PCR, Supplement S5: Protocol for E7 PCR using Hotstar Taq, Figure S1: E7 PCR using Invitrogen Taq, Figure S2: E7 PCR using Hotstar Taq, Figure S3: Comparison of HPV 51+ and β-globin+ results, Figure S4: Proportion of samples positive stratified by cytological grade, Figure S5: Proportion of samples positive stratified by age, Figure S6: Proportion of samples positive stratified by Social Deprivation Score, Figure S7: Proportion of infections in multiple samples. genotyping results were used to compare all multiples, GP5+/6+ PCR EIA + repeat negative multiples and E7 negative multiples, Figure S8: Example of sequencing data for one sample; shows a 99% match to known HPV 51 E7 region.
