# Supplementary material for: High Prevalence of HPV 51 in an Unvaccinated Population and Implications for HPV Vaccines
Source: Vaccines (Basel). 2022 Oct 20;10(10):1754. doi: 10.3390/vaccines10101754 (PMC9611345; doi:10.3390/vaccines10101754)
Supplement: Supplementary file 1 [file vaccines-10-01754-s001.zip › vaccines-1956338-supplementary.pdf]

Supplementary material: High prevalence of HPV 51 in an unvaccinated population and implications for HPV vaccines

Bowden *et al* 2022

Supplement Methods

Supplement S1 – Protocol for DNA Extraction

Each LBC sample was washed and re-suspended in 500µl 10 mM Tris pH 7.4. For every 22 samples, one positive control of 250µl Plasmid 51 (Deutsches Krebsforschungszentrum, Heidelberg) and one negative control of 250µl H20 were created. 50µl of recombinant proteinase K was added to a 250µl cell suspension from each sample and incubated at 56°C overnight with shaking. Samples were then incubated at 90°C for 10min, placed in racks (previously chilled to -20°C) in a 4°C fridge for 10min, centrifuged at 13 000 r.p.m, 4°C, for 10 min and the supernatant transferred into a 96-well plate.

Supplement S2 – Protocol for GP5+/6+ PCR ELISA

Samples were processed in batches of 22 and a two-tier method applied: (i) an initial PCR- ELISA with a cocktail of HR type-specific probes (ii) A second PCR-ELISA using the individual HPV 15 probe. PCR cycling conditions were 94°C – 4mins, (94°C – 30s, 40°C – 90s, 72°C – 60s; x 40), 72°C – 4mins, 15°C – hold). Positive (Plasmid) and negative (water) DNA extraction, PCR and ELISA controls were included for every 22 samples. The final negative extraction control in each 96-well plate serves as the background reading for which all the other results are compared. A positive result is equivalent to three times background.

Supplement S3 – Protocol for Linear E7 PCR

PCR was performed on extracted DNA for each sample including positive and negative controls. 5µl of DNA was added to 20 µl of PCR reagents (2.5µl 10x Invitrogen® buffer, 2.5µl 2mM dNTPs, 51 linear E7 primer 10µM 2.5µl, Invitrogen® Taq 1U 0.125µl, H2O 12.375µl). PCR cycling conditions were 95°C- 15mins (94°C-30s, 62°C-30s, 72°C-3 min) 72°C-7 min, 4°C-hold).

Supplement S4 – Protocol for Nested E7 PCR

5µl of DNA was added to 20 µl of PCR reagents (2.5µl 10x Invitrogen® buffer, 2.5µl 2mM dNTPs, forward primer 5µM 2.5µl, reverse primer 5µM 2.5µl, MgCl2, 25µM 2.5µl, Invitrogen® Taq 1U 0.1µl, H2O 7.4µl). PCR cycling conditions were 95°C- 15mins (94°C- 30s, 58°C-30s, 72°C-30s) 72°C-5 minutes, 4°C-hold). Electrophoresis was performed on E7 PCR products. Results were photographed and compared to a 100bp DNA Ladder to determine positive or negative.

Supplement S5 – Protocol for E7 PCR using Hotstar Taq

5µl of DNA was added to 20 µl of PCR reagents (2.5µl hotstar buffer, 2.5µl 2mM dNTPs, forward primer 5µM 2.5µl, reverse primer 5µM 2.5µl, Hotstar Taq 1U 0.125µl, H2O 9.875µl). PCR cycling conditions were 95°C- 15mins (94°C – 30s, 58°C-30s, 72°C- 30s) 72°C- 5 minutes, 4°C – hold). Electrophoresis was then performed on E7 PCR products. Results were photographed and compared to a 100bp DNA Ladder and results are then given as positive (1) or negative (0) and entered into an Excel worksheet.

Supplementary material: High prevalence of HPV 51 in an unvaccinated population and implications for HPV vaccines

Bowden *et al* 2022

Figures

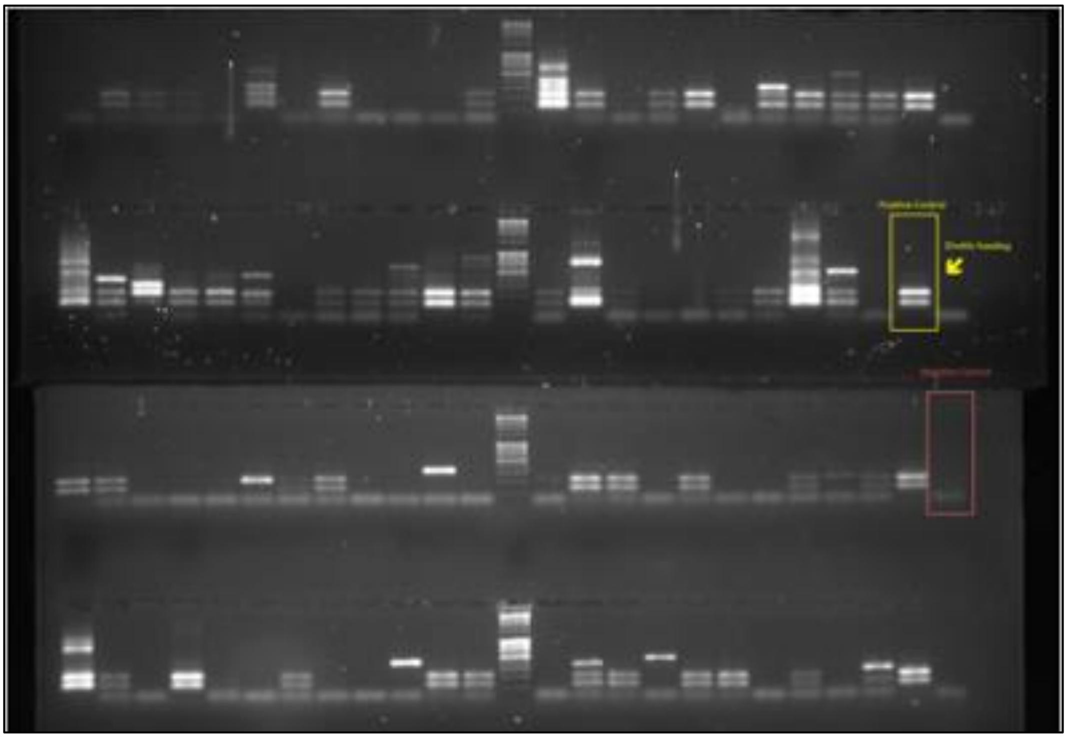

Figure S1: E7 PCR using Invitrogen Taq. Yellow box highlights the double banding in positive samples showing an unexpected fragment.

Supplementary material: High prevalence of HPV 51 in an unvaccinated population and implications for HPV vaccines

Bowden *et al* 2022

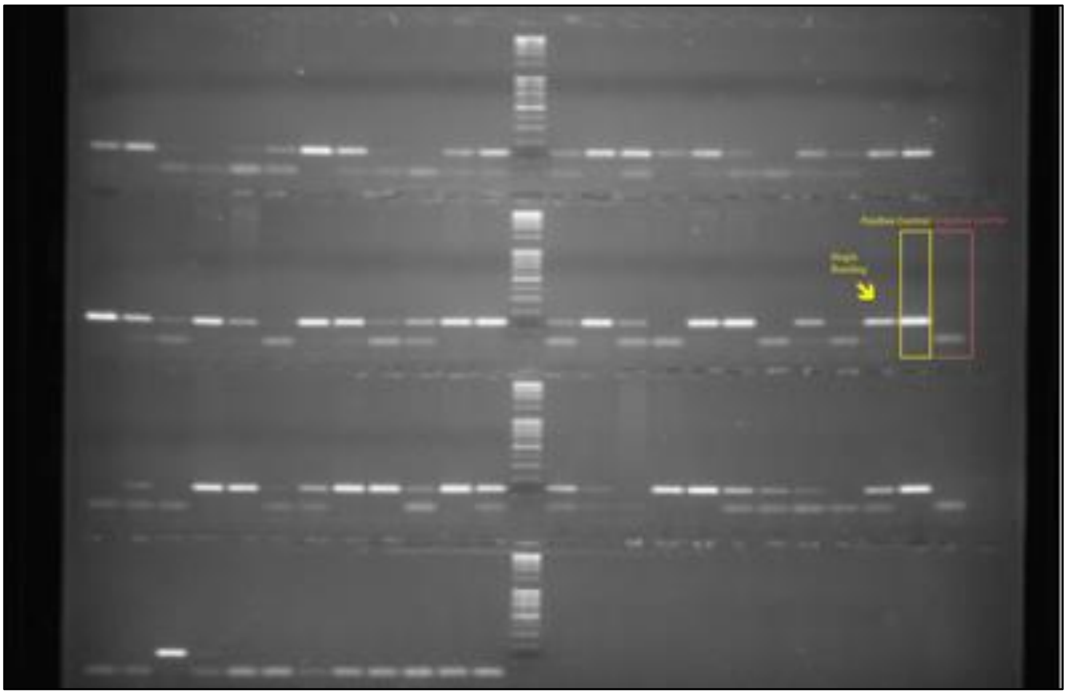

Figure S2: E7 PCR using Hotstar Taq. Yellow box highlights the single band of expected fragment only.

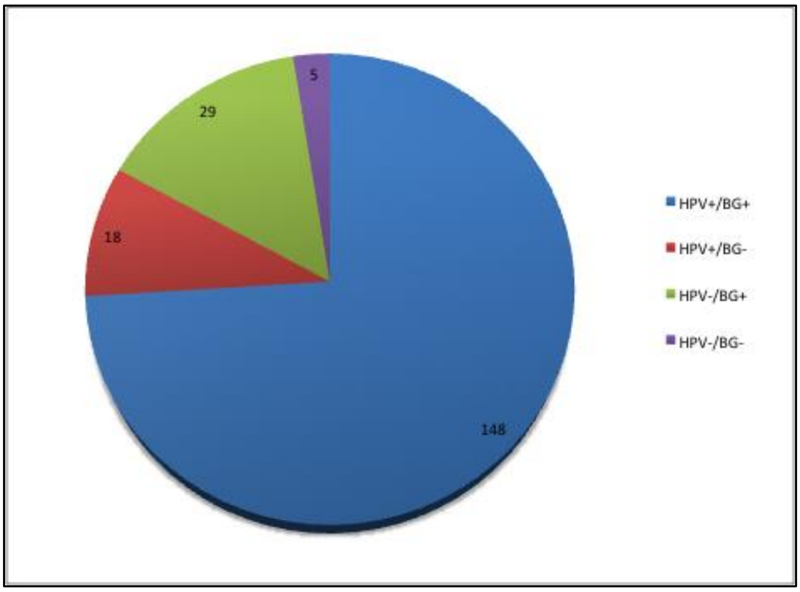

Figure S3: Comparison of HPV 51+ and β-globin+ results

Supplementary material: High prevalence of HPV 51 in an unvaccinated population and implications for HPV vaccines

Bowden *et al* 2022

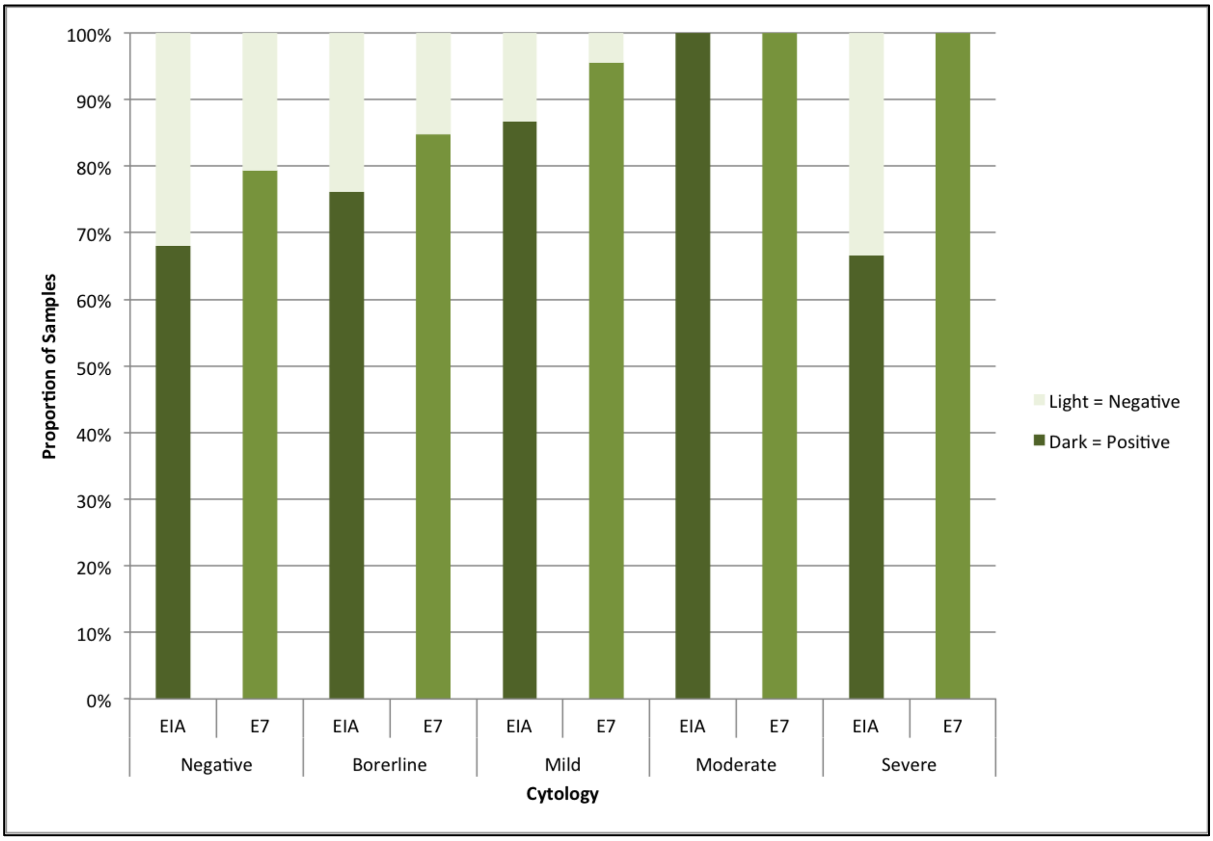

Figure S4. Proportion of samples positive stratified by cytological grade

Supplementary material: High prevalence of HPV 51 in an unvaccinated population and implications for HPV vaccines

Bowden *et al* 2022

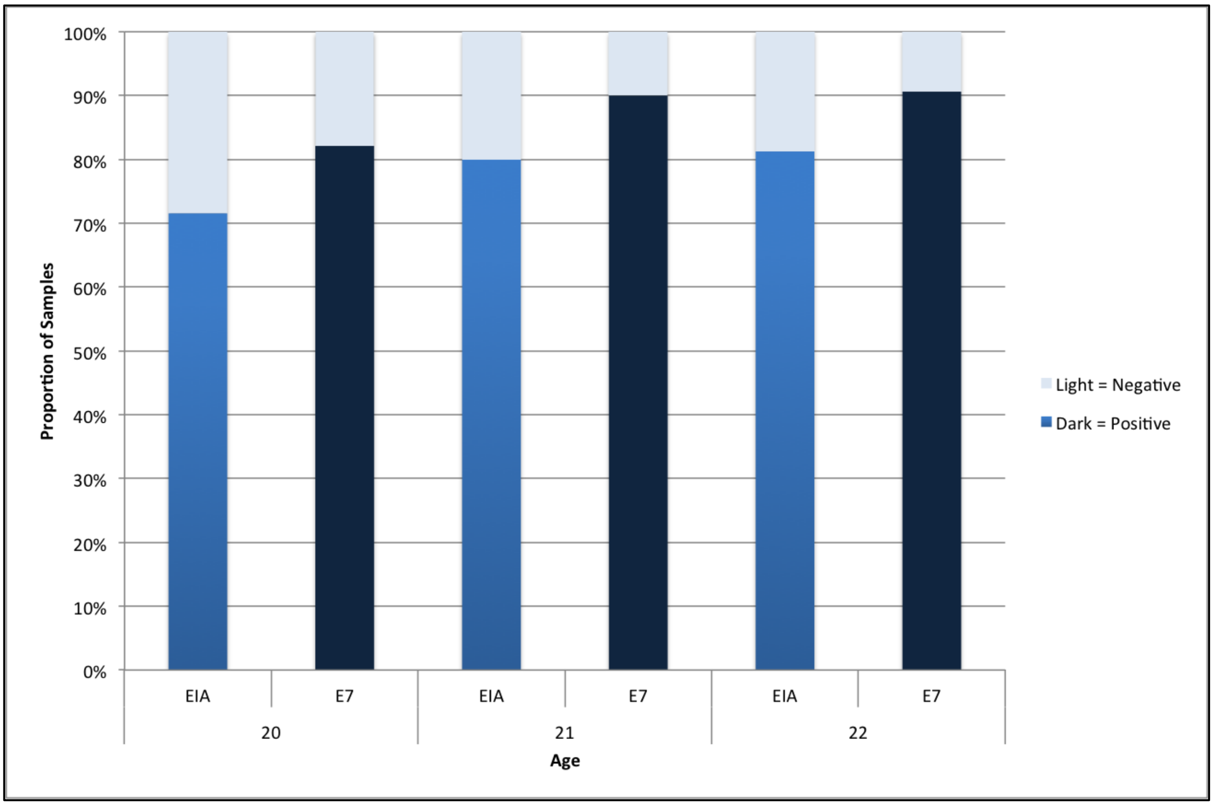

Figure S5. Proportion of samples positive stratified by age

Supplementary material: High prevalence of HPV 51 in an unvaccinated population and implications for HPV vaccines

Bowden *et al* 2022

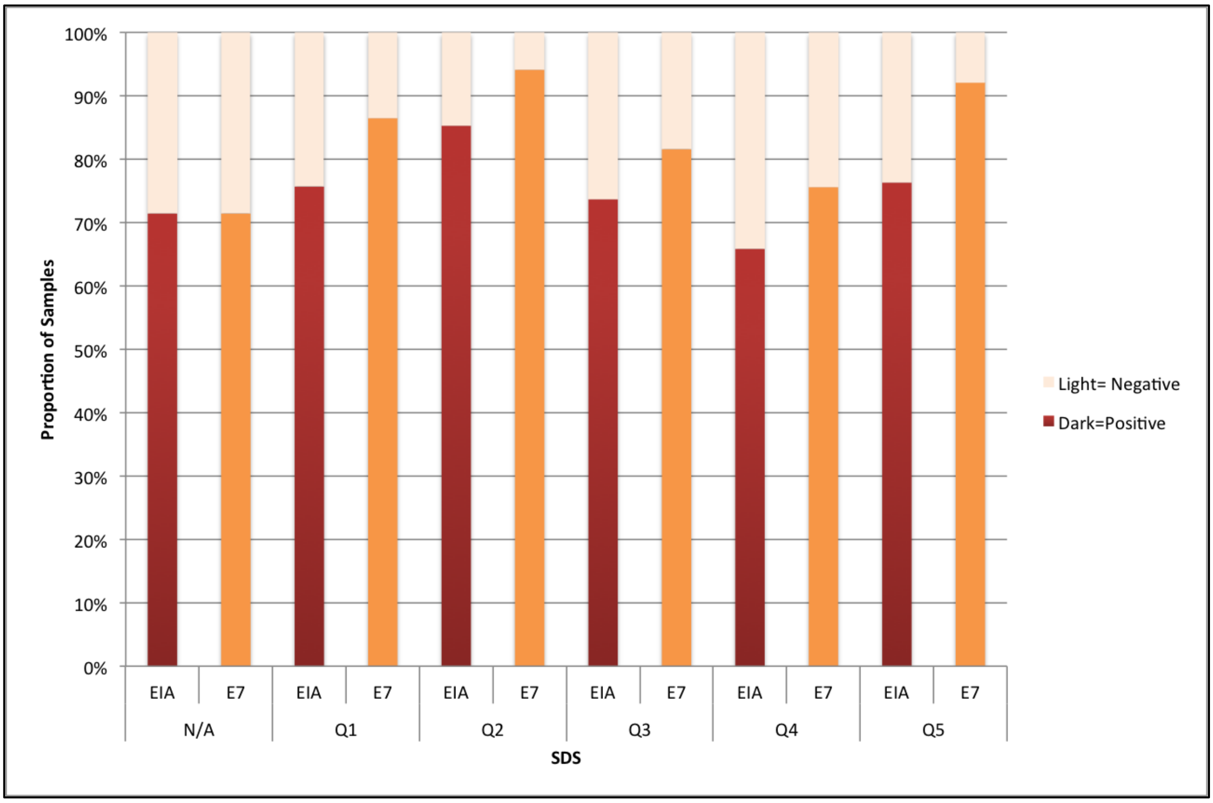

Figure S6. Proportion of samples positive stratified by Social Deprivation Score.

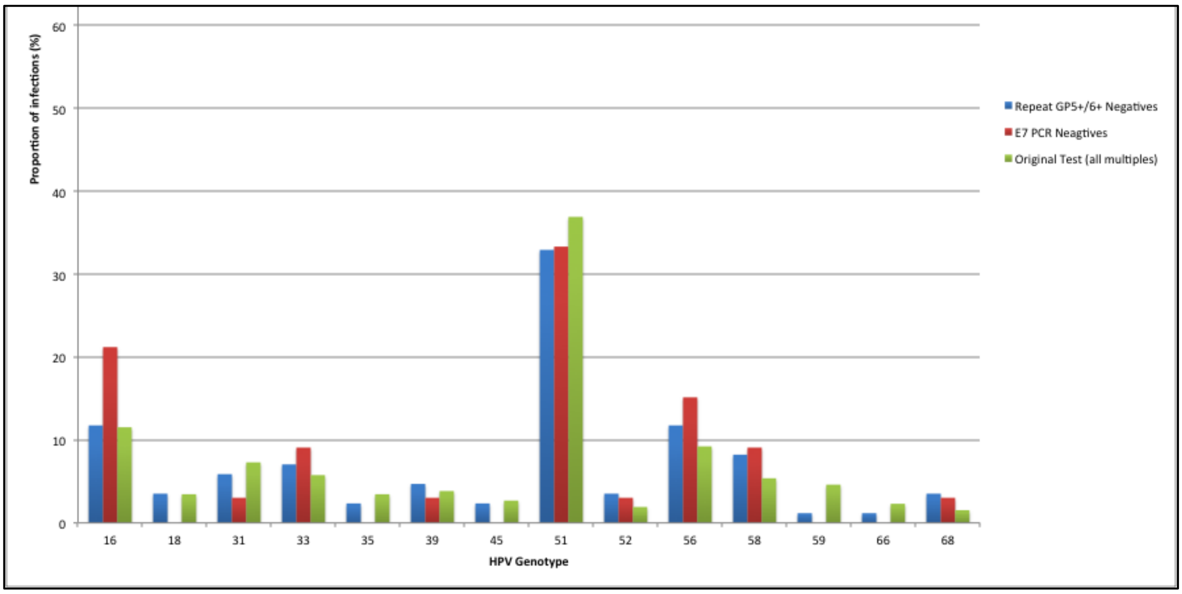

Figure S7. Proportion of infections in multiple samples. Typing results from Base HPV 2009 used to compare all multiples, GP5+/6+ repeat negative multiples and E7 negative multiples.

Bowden *et al* 2022

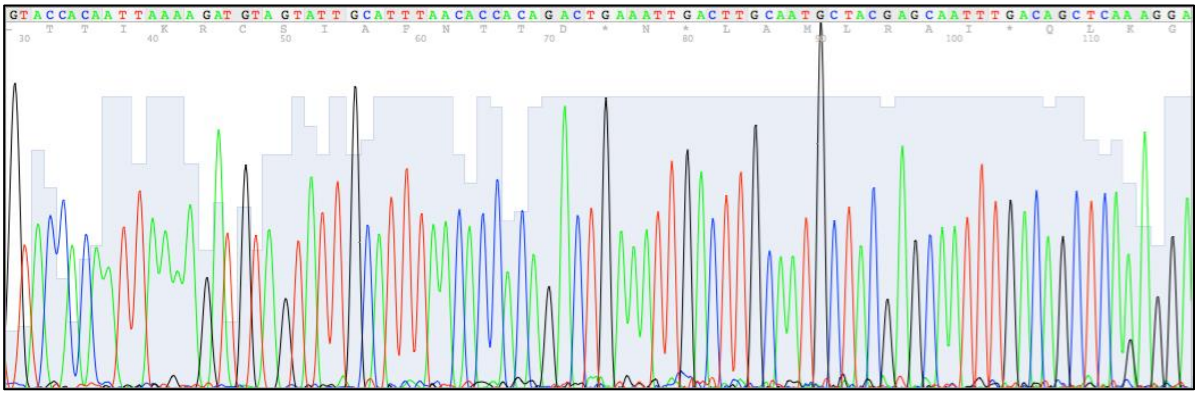

Figure S8. Example of sequencing data for one sample; showing a 99% match to known HPV 51 E7 region.
